# Green and Scalable Synthesis of Efficient and Stable CsPbBr_3_ Perovskite Quantum Dots Enables Low‐Threshold Amplified Spontaneous Emission

**DOI:** 10.1002/advs.76783

**Published:** 2026-07-27

**Authors:** Yongfeng Liu, Mengting Zhang, Qingyu Xie, Kehao Hu, Bowen Zhang, Zhe Zhang, Jie Yang, Zhiping Hu, Juan Du, Jia Wang, Min Zhou

**Affiliations:** ^1^ College of Physical Science and Technology Yangzhou University Yangzhou China; ^2^ School of Physics and Optoelectronic Engineering Hangzhou Institute for Advanced Study University of Chinese Academy of Sciences Hangzhou Zhejiang China; ^3^ College of Physics and Optoelectronic Engineering Chongqing Normal University Chongqing China; ^4^ Department of Physics Umeå University Umeå Sweden; ^5^ Wallenberg Initiative Materials Science for Sustainability, Department of Physics Umeå University Umeå Sweden

**Keywords:** Amplified spontaneous emission, Auger effect, Ostward ripening, perovskite, photoluminescence, quantum dot, quantum yield, Spontaneous emission

## Abstract

Halide perovskite quantum dots (PeQDs) are promising as gain materials for amplified spontaneous emission (ASE), yet their environmentally friendly and scalable wet‐chemistry synthesis remains challenging. Meanwhile, severe nonradiative‐recombination channels impair ASE performance of neat PeQDs. Herein, we demonstrate a green and scalable strategy employing bio‐sourced zwitterionic lecithin as a ligand and hexane as a solvent. This approach enables single‐batch production of high‐quality CsPbBr_3_ PeQDs of over 10 grams, with potential scaling‐up using industrial equipment. Theoretical calculations and experimental characterizations reveal that such scalability stems from suppressed Ostwald ripening, driven by the synergistic covalent and electrostatic interactions between PeQD surface and the functional groups. The resulting PeQDs exhibit remarkably inhibited nonradiative recombination from both Auger recombination and electron‐phonon coupling, alongside a high photoluminescence quantum yield of 95%. Lecithin also endows PeQDs with enhanced stability under air, UV irradiation, heat, and polar solvents. Benefiting from such merits, lecithin‐PeQDs achieve outstanding ASE performance with significantly low thresholds of 230.4 and 50.1 µJ cm^−^^2^ under nanosecond and femtosecond laser excitation, respectively, both representing state‐of‐the‐art levels for neat PeQD films. This work not only provides a green and efficient route for mass production of PeQDs, but opens a new avenue for designing high‐performance gain materials.

## Graphical abstract

Green and scalable synthesis of CsPbBr_3_ perovskite quantum dots using bio‐sourced lecithin and hexane enables single‐batch production over 10 grams. Suppressed Auger recombination and electron‐phonon coupling yields 95% PLQY, enhanced stability, and low ASE thresholds of 230.4 (ns) and 50.1 (fs) µJ cm^−^^2^ in neat films.

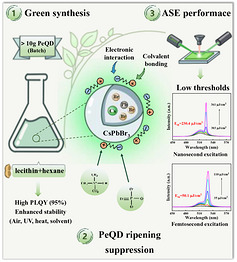

## 1 Introduction

Amplified spontaneous emission (ASE) has garnered significant attention across diverse fields such as integrated photonics, biomedical imaging, and next‐generation displays. This is primarily due to the unique combination of high brightness and spectral purity of lasers with the structural simplicity and high integrability of cavity‐free systems [1]. Among the various gain materials explored for ASE, all‐inorganic lead halide perovskite materials have distinguished themselves through their exceptional optical properties, including their high photoluminescence quantum yield (PLQY), tunable bandgap, and excellent solution processability [2, 3, 4, 5, 6, 7]. While perovskite polycrystalline films have achieved significant progress in ASE applications [8, 9], they remain plagued by severe non‐radiative recombination at grain boundaries, poor long‐term stability, and challenges in reproducible synthesis [10, 11, 12, 13]. In contrast, perovskite quantum dots (PeQDs), benefiting from advanced surface ligand engineering, offer superior optical characteristics including near‐unity PLQY, suppressed non‐radiative recombination, and high colloidal stability [14], positioning them as a more robust platform for solution‐processable gain media [15, 16].

The practical deployment of PeQD‐based ASE devices necessitates synthetic methodologies that are both environmentally benign and industrially scalable. Currently, the synthesis of PeQDs predominantly follows two routes: hot‐injection (HI) and ligand‐assisted reprecipitation (LARP). The HI method achieves burst nucleation at elevated temperatures, yielding QDs with uniform size and high crystallinity; [17, 18] however, its stringent requirements for inert atmospheres, precise temperature control, and skilled operation render it economically prohibitive and difficult to scale. The LARP method, conducted at room temperature, offers greater potential for industrial translation due to its operational simplicity and lower energy consumption [19, 20], as also demonstrated by our prior work through a scaled‐up LARP synthesis of 1.8 g PeQDs with near‐unity PLQY [21]. Nevertheless, conventional LARP approaches rely heavily on toxic aromatic solvents such as toluene to trigger nucleation via polarity shifts—a process fundamentally incompatible with green chemistry principles. Moreover, existing gram‐scale syntheses remain insufficient for industrial adoption, and the colloidal stability and non‐radiative recombination channels of such PeQDs require substantial improvement for high‐performance ASE.

Since the first report of ASE in MAPbI_3_ thin films, perovskite‐based gain media have witnessed rapid development [22]. While high‐performance perovskite ASE has been achieved through optical microcavities, such as whispering gallery modes [23, 24, 25, 26, 27], distributed feedback gratings [28, 29, 30, 31, 32], or vertical‐cavity surface‐emitting lasers [33, 34, 35], these architectures often require complex fabrication and offer limited spectral tuning flexibility [36]. Alternatively, cavity‐free neat PeQD films have been an ideal candidate due to simplified processing and facile wavelength tuning; however, their performance now is still hampered by intrinsic non‐radiative recombination channels, particularly Auger recombination and electron‐phonon coupling, which quench optical gain through competing with stimulated emission, compromising ASE performance [37, 38]. Therefore, it is still a critical yet unmet challenge to develop massive synthesis of high‐quality PeQDs through a green route. It should be noted that lecithin and other zwitterionic ligands have been previously used in PeQDs. For example, Krieg et al. reported improved durability of CsPbX_3_ PeQDs using zwitterionic ligands [39]. Mir et al. demonstrated ultrastable CsPbI_3_ PeQDs capped with lecithin for LED applications [40]. In 2023, Stefania Milanese and colleagues proposed that lecithin could be used in the field of ASE; [41] in 2025, the same team achieved an ASE threshold of 300 µJ/cm^2^ using lecithin‐modified quantum dots, but the ASE stability remained poor (only 10% retention after 120 min) [42]. Notably, most of the previous reports on lecithin or zwitterion‐capped PeQDs relied on the hot‐injection method, which requires high temperatures, an inert atmosphere, and complex procedures, making it less environmentally friendly and difficult to scale up. In contrast, our synthesis is conducted at room temperature using hexane as a solvent, offering a relatively environmentally friendly, scalable, and operationally simple alternative.

Herein, we report a scalable synthesis strategy that employs bio‐sourced zwitterionic lecithin as a capping ligand and hexane—a low‐polarity solvent—as the reaction medium. Compared with conventional aromatic solvent systems (e.g., toluene) widely used in CsPbBr_3_ PeQD synthesis and processing, this hexane‑based route reduces the reliance on aromatic solvents. In addition, the use of lecithin, a bio‑derived, low‑cost, and readily available phospholipid ligand, provides sustainability advantages from the ligand‑design perspective. Collectively, this combined solvent–ligand design offers a relatively greener alternative. Density functional theory (DFT) calculations and in situ photoluminescence (PL) spectroscopy reveal that the synergistic covalent‐electrostatic interaction between lecithin and the PeQD surface effectively suppresses Ostwald ripening. This allows for scalable synthesis of 10 g via one batch. The resulting PeQDs exhibit a high PLQY of ∼95%, inhibited Auger and electron–phonon coupling losses, and exceptional stability under ambient, thermal, UV, and polar solvent exposure. Consequently, these neat PeQD films achieve remarkably low ASE thresholds of 230.4 µJ cm^−^^2^ (nanosecond) and 50.1 µJ cm^−^^2^ (femtosecond) without any external optical cavity. This work provides a relatively greener and scalable synthesis route for PeQDs and opens a new avenue for designing high‐performance gain media suitable for practical coherent light sources.

## 2 Results and Discussion

### 2.1 Calculation and Scalable Synthesis

To achieve green and scalable synthesis, here we employ hexane as a reaction medium due to its low toxicity, high dispersing ability to PeQDs, and cost‐effectiveness [43], while using lecithin as a passivation ligand, which offers multiple advantages in room‐temperature large‐scale synthesis. (1) Lecithin possesses a zwitterionic structure, comprising a positively charged choline group and a negatively charged phosphate group. This bipolarity enables simultaneous passivation of both cationic and anionic defects on the PeQD surfaces; (2) its two branched alkyl chains (C16 and C18) not only ensure superior dispersibility in nonpolar solvents, such as hexane, but also contribute to colloidal stability by providing steric hindrance; (3) it is commercially available and cost‐effective (2.2 CNY/g); (4) as a natural phospholipid, lecithin is nontoxic and biodegradable, aligning with the development of sustainable materials for optoelectronic applications.

**FIGURE 1:**
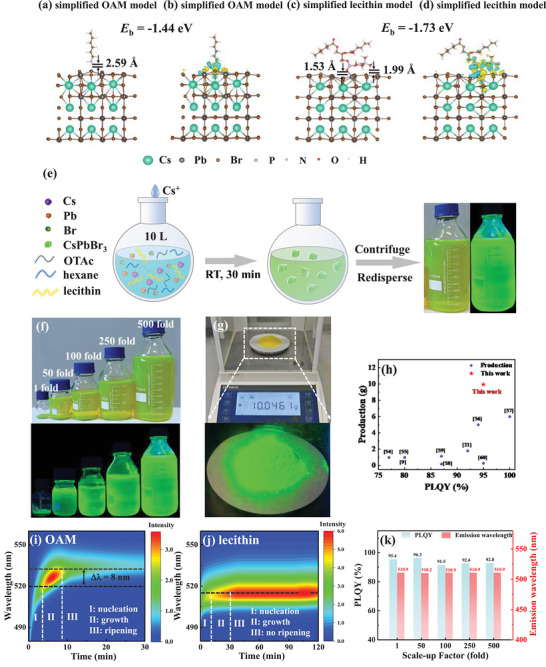
Binding mode of (a) simplified OAM model and (c) simplified lecithin model on the CsPbBr_3_ perovskite surface. Differential charge density after adsorption of (b) the simplified OAM model and (d) simplified lecithin model, where yellow and blue regions denote charge accumulation and depletion, respectively. (e) Schematic diagram of the LARP synthesis procedure for CsPbBr_3_ PeQDs. (f) Lecithin‐PeQDs under daylight and UV light. (g) Photograph of as‐obtained lecithin‐PeQDs powder (≈10.0 g) from 500‐fold scale‐up synthesis at 30 min. (h) Summary of PLQY and production for CsPbX_3_ PeQDs from liquid‐phase synthesis in different ligand systems from the literature. In situ PL monitoring of (i) OAM‐PeQDs and (j) lecithin‐PeQDs. (k) Statistical distribution of PLQY and emission peak positions for lecithin‐PeQDs at different reaction scales.

To investigate the feasibility of the lecithin ligand in suppressing Ostwald ripening in CsPbBr_3_ PeQDs [44], we thus theoretically investigated the binding behavior of oleylamine (OAM) and lecithin on CsPbBr_3_ perovskite slab surfaces using DFT calculations [45]. In the CsPbBr_3_‐ligand model, a PbBr_2_‐terminated surface was utilized with uncoordinated Pb^2+^ and Br^–^ ions. To achieve maximum calculation efficiency without sacrificing accuracy or relevance [46], here we simplify the ligands by decreasing the alkyl chain length of both ligands from 18/16 to 6, namely the simplified OAM model and the simplified lecithin model (Figures S1 and S2). Figure 1a indicates that the simplified OAM model interacts with the CsPbBr_3_ surface primarily via strong electrostatic attraction between its protonated amine NH_3_^+^ from the ligand and Br^−^ from the perovskite interface, with a distance (Br–N) of 2.58 Å [47]. Besides, the simplified OAM model ligand lies almost perpendicularly to the perovskite surface via physical adsorption [48], and presents weak binding capacity to the perovskite slab, as verified by a small binding energy (*E*_b_) of −1.44 eV calculated using Equation (S1). In contrast, the simplified lecithin model exhibits a significantly different scenario: the oxygen atom in its phosphate group forms a stable Pb─O covalent bond with the undercoordinated Pb^2^^+^ on the surface, while the quaternary ammonium group engages in strong electrostatic interactions with surface Br^−^ ions, forming a bidentate binding configuration (Figure 1c) [49]. This multi‐site bonding mode could substantially enhance the anchoring strength between the ligand and the substrate [50]. The Pb─O and Br─N distances in the simplified lecithin model system are 1.53 Å and 1.99 Å, respectively, the latter of which is much shorter than that of the simplified OAM model system, inferring stronger binding ability [51]. Furthermore, the simplified lecithin model exhibits a significantly stronger *E*_b_ of −1.73 eV, reflecting that lecithin could tightly bind to the perovskite surface and is beneficial to PeQDs’ optoelectronic properties and to improving tolerance to external stimuli.

Further electronic structure analysis reveals that the introduction of lecithin markedly influences the electronic properties of CsPbBr_3_ PeQDs. The projected density of states (PDOS) results (Figure S3) show that the band edges of CsPbBr_3_ are primarily composed of Pb 6p and Br 4p orbitals. Upon lecithin introduction, the Pb 6p orbital shifts noticeably upward near the Fermi level, and the band gap narrows from 2.10 to 2.05 eV. This change suggests strong coordinative interaction between the phosphate group and surface Pb^2^^+^, effectively modulating the interfacial electronic structure [52]. Charge density difference analysis provides direct electronic evidence for the distinct binding modes of the two ligands. In the simplified OAM model system (Figure 1b), only localized and weak electron redistribution occurs between the ammonium group (NH_3_^+^) and surface Br^−^ ions, with no significant charge transfer, which is consistent with a physical adsorption mechanism [53]. In sharp contrast, the simplified lecithin model system (Figure 1d) exhibits pronounced electron depletion (blue) around undercoordinated Pb^2^^+^ sites and clear electron accumulation (yellow) around the phosphate oxygen atoms, indicating directed Pb‐to‐O charge transfer and confirming a stable Pb–O covalent bond. Furthermore, the quaternary ammonium group of lecithin engages in much stronger and more extensive electron redistribution with surface Br^−^ ions than in the simplified OAM model case. This distinct electronic contrast aligns well with the notably higher binding energy of the simplified lecithin model, confirming that its anchorage is stabilized via synergistic covalent–electrostatic interactions, rather than the physical adsorption predominant in the simplified OAM model system. Based on the combined DFT calculations, the strong bonding and bidentate interaction formed between lecithin and the PeQD surface is expected to effectively suppress ligand desorption and alleviate its dynamic binding, and thus provide a theoretical basis for inhibiting Ostwald ripening during large‐scale synthesis. In combination with its alignment of natural and biodegradable properties with the green‐synthesis principles, we thus anticipate that the lecithin ligand could enable the green and scalable production of high‐performance PeQDs.

Guided by the theoretical calculations, a co‐capping ligand system, comprising octanoic acid (OTAc) and OAM/lecithin, is designed (Figure S1). This design rationale considers that OTAc improves lecithin's solubility in the reaction solvent and PeQDs stability in hexane, while lecithin's zwitterionic structure enables simultaneous passivation of both cationic and anionic defects. Based on this theoretical framework, we synthesized PeQDs using the LARP method at room temperature, but employing green hexane as reaction medium to replace the commonly used and highly toxic DMF or toluene, ensuring scalability and environmental friendliness [43]. The detailed procedure (Figure 1e) involves thorough mixing of precursor solutions containing Cs‐OTAc, Pb‐OTAc in hexane, and lecithin‐OTAc, followed by rapid injection of Br‐OTAc in hexane with a 30‐min reaction. After antisolvent purification, the resulting lecithin‐PeQDs are dispersed in hexane, forming a bright green colloidal solution for characterization. As shown in Figure 1f, the lecithin‐PeQDs exhibit bright green emission under UV light and good transparency under daylight. Control samples (OAM‐PeQDs) were prepared under identical conditions using OAM to replace lecithin, with detailed optimization in Figures S4 and S5. To evaluate the compatibility of the conventional OAM ligand with hexane, we synthesized OAM‑capped PeQDs in hexane (OAM‑PeQDs in hexane) and compared their optical properties with those of OAM‑PeQDs synthesized in toluene (Figure S6). The scale‑up behavior of OAM‑PeQDs was further characterized at different reaction scaling factors (Figures S7–S9), which showed a significant degradation in PLQY and spectral uniformity with increasing scale. The OAM‑PeQDs in hexane exhibited a PLQY of only 48% with the full width at half maximum (FWHM) of 25 nm, significantly lower than that of the toluene‑synthesized counterpart (PLQY ∼ 64%). In contrast, lecithin‑capped PeQDs synthesized directly in hexane achieved a PLQY as high as 95% (see below). These results indicate that the conventional OAM ligand does not work efficiently in hexane at room temperature, likely due to poor ligand solubility or insufficient surface passivation. By contrast, the zwitterionic lecithin ligand is well‑suited for hexane‑based synthesis, enabling high‑quality PeQDs with superior optical performance. Therefore, the use of hexane as a more environmentally friendly alternative to toluene is only effective when combined with the lecithin ligand.

In contrast, lecithin‐PeQDs only exhibit two periods: nucleation (0–10 min), growth (10–30 min), and a stable emission maintained throughout 30–120 min of interest without any change in both PL wavelength and intensity, which manifests the absence of undesired Ostwald ripening. This delayed growth kinetics confirms strong binding dynamics to the PeQD core. Furthermore, the successful large‐scale synthesis is evidenced by the photograph of the as‐obtained lecithin‐PeQDs powder (≈10.0 g) from the 500‐fold scale‐up shown in Figure 1g. A comprehensive comparison with previously reported batch syntheses is presented in Table S1 and illustrated in Figure, which summarizes the key parameters for CsPbBr3 PeQDs prepared via wet‑chemistry routes [9, 21, 54, 55, 56, 57, 58, 59, 60. The in situ PL monitoring results (Figure 1i for OAM‐PeQDs and Figure 1j for lecithin‐PeQDs) further support these kinetic profiles. Lecithin‑PeQDs maintained high PLQY and stable optical properties across different reaction scales (Figure S10). To systematically investigate the temporal effects on the optical properties, lecithin‐PeQDs obtained at selected time intervals (30, 60, 90, 120 min) were further characterized by PL, PLQY, UV–vis absorption spectroscopy, FTIR, x‐ray diffraction (XRD), and transmission electron microscopy (TEM), as shown in Figures S11 and S12. All samples demonstrated high PLQYs exceeding 90% and a stable PL emission peak at 512 nm without spectral shifts. This consistency was further corroborated by the invariant excitonic absorption features in the UV–vis spectra. XRD patterns confirm that all diffraction peaks could be indexed to the monoclinic CsPbBr_3_ phase (PDF#18‐0364), and no phase transformation was induced by prolonged reaction times. TEM statistical analysis reveals a narrow size distribution ranging from 9.18 to 10.13 nm, with the average particle size fluctuating by less than 5%, confirming excellent size uniformity and temporal stability. FTIR spectra showed constant positions of characteristic functional group vibrations (C═O: 1733 cm^−^^1^; P═O: 858 cm^−^^1^; C─H: 2925/2854 cm^−^^1^) from 30 to 120 min, with only minor intensity variations (RSD < 5%). No enhancement of O─H/N─H signals or appearance of impurity‐derived peaks was detected. Photographs of OAM‐PeQDs and lecithin‐PeQDs under daylight at different reaction times also support the above observations. These characterizations confirm that the notorious Ostwald ripening, as expected, is indeed not present in the synthesis process of 500‐fold scale‐up over a 120 min span. These results indicate that the PLQY, production, and production yield of the lecithin system are among the top levels. The statistical distribution of PLQY and emission peak positions for lecithin‐PeQDs at different reaction scales is presented in Figure 1k, demonstrating excellent consistency across scales. To quantify the production efficiency of the scaled‑up synthesis, we calculated the yields for both lecithin‑PeQDs (500‑fold) and OAM‑PeQDs (5‑fold). Table s2 summarizes the weight statistics and yields. The lecithin‑PeQDs exhibit a significantly higher yield (82.5%) compared to OAM‑PeQDs (32.4%), demonstrating the advantage of the lecithin‑assisted synthesis for large‑scale production. In summary, owing to the unique bidentate binding mechanism, lecithin ligand enables green and scalable production of CsPbX_3_ PeQDs by inhibiting Ostwald ripening.

### 2.2 Compositional Analysis

**FIGURE 2:**
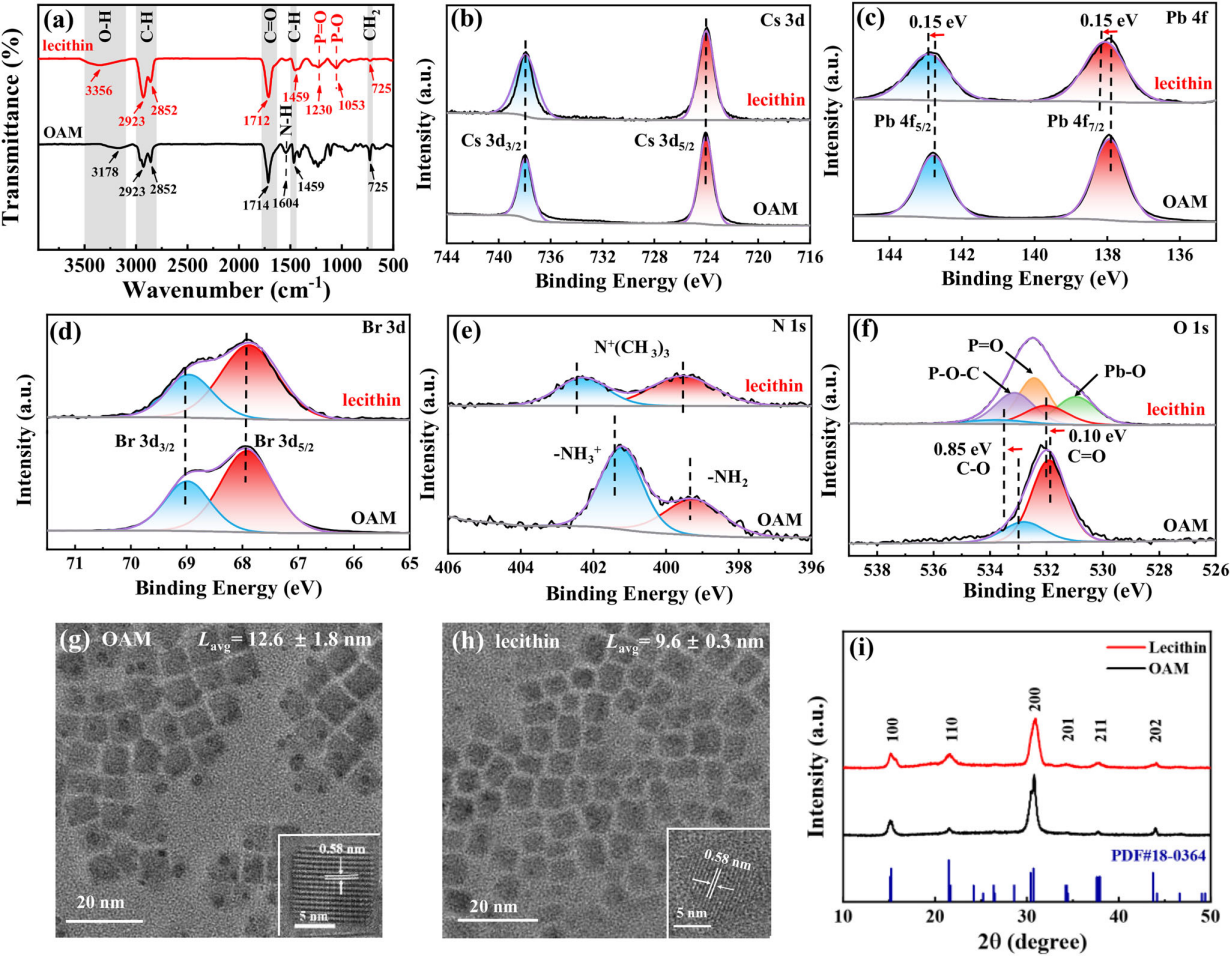
Ligand chemistry and surface analysis of OAM‐PeQDs and lecithin‐PeQDs. (a) FTIR spectra. High‐resolution XPS spectra in the (b) Cs 3d, (c) Pb 4f, (d) Br 3d, (e) N 1s, and (f) O 1s regions. TEM images with insets showing HRTEM images of (g) OAM‐PeQDs and (h) lecithin‐PeQDs. (i) XRD patterns of PeQDs.

To investigate the surface chemistry, Fourier transform infrared (FTIR) spectroscopy was performed. Figure 2a shows that the distinct C═O stretching vibrations at around 1714 cm^−^^1^ in both samples confirm the presence of OTAc ligands on the PeQD surface [61]. In the OAM‐PeQDs spectrum, the weak band at 1604 cm^−^^1^ originates from the N─H bending mode, confirming the coordination of OAM [62]. In contrast, the lecithin‐PeQDs spectrum exhibits characteristic peaks at 1230 and 1053 cm^−^^1^, which are assigned to the P═O and P─O─C stretching vibrations from the phosphate group of lecithin, respectively [63]. The strong absorptions at 2923, 2852, and 1459 cm^−^^1^ are attributed to the asymmetric stretching, symmetric stretching, and bending vibrations of C─H bonds, respectively [61], while the weak band at 725 cm^−^^1^ corresponds to the in‐plane rocking vibration of ─CH_2_– groups. It is noted that both samples display O─H stretching vibrations at 3336 and 3178 cm^−^^1^, which arise from residual water in the atmosphere [64]. These results collectively demonstrate the successful anchoring of lecithin onto the PeQD surface via ligand exchange.

The surface chemical compositions of the PeQDs were systematically analyzed by x‐ray photoelectron spectroscopy (XPS). Figures 2b–f and Figure S13 present the high‐resolution XPS spectra of the constituent elements in OAM‐PeQDs and lecithin‐PeQDs powders. The XPS survey spectra (Figure S14) confirm the presence of Cs, Pb, Br, C, N, and O in OAM‐PeQDs, while lecithin‐PeQDs exhibit a distinct additional P signal, confirming their respective compositions. Quantitative elemental analysis (Table S3) indicates that the Cs:Pb:Br atomic ratio in OAM‐PeQDs is 1.1:1:3.4, close to the theoretical stoichiometry of CsPbBr_3_. In contrast, lecithin‐PeQDs exhibit a markedly Cs‐ and Br‐enriched surface (1.8:1:3.6). To provide a quantitative comparison with the literature, we have summarized representative Cs:Pb:Br ratios of CsPbBr_3_ PeQDs from previous reports in Table S4. Our OAM‑PeQDs ratio (1.1:1:3.4) shows a deviation of +10% for Cs and +13% for Br from the nominal 1:1:3, while the lecithin‑PeQDs ratio (1.8:1:3.6) deviates by +80% for Cs and +20% for Br. The OAM‑PeQDs ratio (1.1:1:3.4) falls within the range of literature‑reported ratios (spanning from 1:1:3.4 to 1.5:1:3.9), reflecting the diversity of surface compositions arising from different ligand systems and synthesis conditions. In contrast, the lecithin‑PeQDs ratio (1.8:1:3.6) exceeds this range, which we attribute to the strong surface enrichment induced by the phospholipid ligand. It is noteworthy that the Cs and Br enrichment is recognized to facilitate effective defect passivation [65]. Further analysis of characteristic elements reveals that the N/Pb ratio is 1.5 in OAM‐PeQDs, while it increases to 3.1 in lecithin‐PeQDs, accompanied by a P/Pb ratio of 2.7. The resulting N:P ratio of approximately 1.1:1 from lecithin‐PeQD aligns well with the theoretical N:P stoichiometry of lecithin (1:1), indicating that lecithin anchors onto the PeQD surface as intact molecules and demonstrating the accuracy of our data.

High‐resolution XPS spectra further elucidate the ligand‐surface interactions. The high‐resolution XPS spectrum of Cs 3d (Figure 2b) confirms the presence of cesium, consistent with the perovskite structure. In the Pb 4f region (Figure 2c), the binding energy of lecithin‐PeQDs shifts by approximately 0.15 eV toward higher energy compared to OAM‐PeQDs, indicating a significant change in the chemical environment around Pb^2^^+^ due to electron transfer from Pb^2+^ to lecithin, in line with the theoretical calculations. This further confirms that the phosphate groups in lecithin effectively passivate under‐coordinated Pb sites via the formation of P─O─Pb bonds. The Br 3d spectrum (Figure 2d) confirms the presence of bromide, also in agreement with the expected perovskite composition. The N 1s spectrum (Figure 2e) clearly reflects the distinct surface coordination chemistry of the two PeQD types: OAM‐PeQDs show two peaks at 399.3 eV and 401.3 eV, corresponding to the amino (‐NH_2_) and protonated ammonium (‐NH_3_^+^) groups, with relative proportions of 36.5% and 63.9%, respectively [66]. Upon lecithin modification, the N 1s peaks of the lecithin‐PeQDs shift to higher binding energies of 400.7 eV and 402.3 eV, corresponding to both the characteristic quaternary ammonium groups (‐N(CH_3_)_3_^+^) of the lecithin molecule. These results not only confirm the successful replacement of OAM by lecithin but also indicate that the surface chemical environment of the QDs is now dominated by positively charged quaternary ammonium groups. This configuration provides a more stable and charge‐definite surface structure, which is conducive to more effective surface passivation [67]. The O 1s spectrum (Figure 2f) provides further direct evidence of the coordination structure: OAM‐PeQDs can be deconvoluted into two components at 531.9 eV (C═O) and 532.8 eV (C─O), originating from the two oxygen species in the carboxyl group of OTAc, whereas lecithin‐PeQDs show a distinct P─O─Pb bonding feature at 530.95 eV, along with P═O and P─O structures of the lecithin phosphate group at 532.45 eV and 533.65 eV, respectively, confirming the strong interaction between lecithin and the PeQD core. Moreover, the introduction of P═O and P─O components leads to a reduction in intensity and a shift toward higher binding energy for the original C═O and C─O signals, reflecting a redistribution of the electronic environment among ligands [68]. The P 2p spectrum (Figure S12) displays a characteristic doublet at 133.15 eV and 134.00 eV, further verifying the successful anchoring of lecithin. Collectively, these systematic XPS analyses demonstrate that lecithin directs the formation of an ultra‐dense, strongly coordinated surface ligand layer, providing atomic‐level insight into the mechanism underlying the long‐term stability of this system under green, large‐scale synthesis conditions.

To investigate the influence of lecithin on the morphology and crystal structure of PeQDs, TEM analysis was conducted. In Figure 2g,h, the TEM results reveal distinct morphological differences: OAM‐PeQDs exhibit a cubic shape with an average edge length (*L_avg_*) of 12.6 ± 1.8 nm, whereas lecithin‐PeQDs adopt a quasi‐spherical morphology with a significantly reduced *L*_avg_ of 9.6 ± 0.3 nm (Figure S15). This difference is attributed to the enhanced steric hindrance effect and improved binding ability of lecithin, which acts as a bidentate ligand to effectively regulate the PeQD growth kinetics. Furthermore, the standard deviation of particle size decreased dramatically from 1.8 to 0.3 nm, indicating a remarkable improvement in size uniformity for lecithin‐PeQDs. The high‐resolution transmission electron microscope (HRTEM) images, provided as insets of Figure 2g,h, further demonstrate the high crystallinity of both samples. Clear lattice fringes with an interplanar spacing of 0.58 nm were observed, corresponding to the (100) crystallographic plane of the cubic perovskite phase. The XRD patterns in Figure 2i for both OAM‐PeQDs and lecithin‐PeQDs powders are indexed to the monoclinic CsPbBr_3_ phase, and confirm that the ligand modification does not compromise the intrinsic crystal structure of the PeQDs [69].

Quantitative surface ligand analysis reveals distinct differences in surface chemistry between the control (OAM‐PeQDs) and experimental (lecithin‐PeQDs) systems. Based on combined thermogravimetric analysis (TGA) (Figure S16), TEM, and XPS analysis (detailed calculation in Equation S2), the total surface ligand density for OAM‐PeQDs is 4.8 molecules nm^−^^2^ (OAM: 3.1 nm^−^^2^, OTAc: 1.7 nm^−^^2^). In contrast, the total density for lecithin‐PeQDs is 2.2 molecules nm^−^^2^ (lecithin: ≈0.8 nm^−^^2^, OTAc: ≈1.1 nm^−^^2^), both of which are lower (Table S5). This quantitative result, together with our earlier binding energy calculations (lecithin: −1.73 eV; OAM: −1.33 eV), supports the core mechanism of this study: the zwitterionic ligand lecithin in the experimental system passivates the surface via a “less dense but stronger” covalent–electrostatic synergistic anchoring (fewer ligands per unit area with higher individual binding energy), whereas the control system relies on a ‘more abundant but weaker’ physical adsorption layer (higher ligand density with weaker interactions). This fundamental difference in surface binding mode microscopically explains the superior performance of the experimental PeQDs in suppressing Ostwald ripening and enhancing stability.

### 2.3 Optical Properties

**FIGURE 3:**
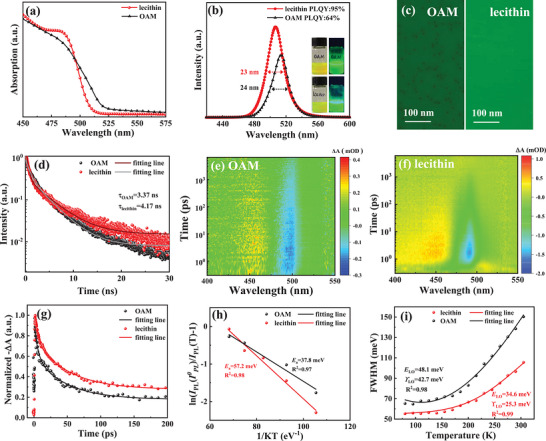
Optical properties of OAM‐PeQDs and lecithin‐PeQDs. (a) UV–vis absorption and (b) PL spectra under 365 nm excitation; (c) PL mapping of OAM‐PeQDs and lecithin‐PeQDs films. (d) TRPL decay curves of OAM‐PeQDs and lecithin‐PeQDs. Representative pseudo‐color TA spectra of (e) OAM‐PeQDs films and (f) lecithin‐PeQDs films. (g) Normalized band‐edge GSB kinetics of both samples. (h) Linear dependence of ln[*I*_PL_($I_{PL}^{0}$)/*I*_PL_(T)‐1] on 1/*k*_B_ *T*, and (i) fitting results of the FWHM as a function of temperature, extracted from their temperature‐dependent PL (Figure S21).

Subsequently, a systematic investigation was conducted to characterize the influence of the lecithin ligand on the optical properties of the PeQDs. Figure 3a,b display the UV–vis absorption and PL spectra of the PeQDs dispersion in hexane solution (∼20 g/L), respectively. The absorption spectra reveal that the emission peak of OAM‐PeQD is located at 518 nm, while that of lecithin‐PeQDs is blue‐shifted to 510 nm. More importantly, the absorption peak of lecithin‐PeQDs exhibits a sharper profile, indicating a more well‐defined excitonic absorption feature [70]. Figure 3c presents the PL mapping of OAM‐PeQD and lecithin‐PeQD films. The OAM‐PeQD film shows obvious voids and defects, whereas the lecithin‐PeQD film exhibits a uniform, bright, and void‐free emission. When transferred to film status, the PLQY remains as high as 81.2% for lecithin‐PeQDs, while it drops to only 47.2% for OAM‐PeQDs (Figure S17), consistent with the excellent optical properties observed in the PL mapping (Figure 3c). As expected, the FWHM decreases from 24 to 23 nm, suggesting a more uniform size distribution and a more symmetric PL peak profile for lecithin‐PeQDs. Moreover, the passivation effect of lecithin is universal to both blue‐ and red‐emitting PeQDs (Figures S18 and S19). Under the tested conditions, the absorption features of the PeQDs remain largely unchanged.

To elucidate the mechanism behind the enhanced optical performance, time‐resolved photoluminescence (TRPL) measurements were carried out. Figure 3d shows the TRPL decay curves of OAM‐PeQDs and lecithin‐PeQDs films, which were well fitted with a biexponential function (Equations S3 and S4). The fitted lifetime parameters are summarized in Table S6. Here, the short decay lifetime (*τ*_1_) is attributed to nonradiative recombination via surface defects, while the long decay component (*τ*_2_) represents intrinsic radiative recombination. *A*_1_ and *A*_2_ denote the corresponding amplitudes [71, 72]. The average lifetime (*τ*_avg_) was calculated using Equation (S4). Compared with OAM‐PeQDs (*τ*_avg_ = 3.37 ns), lecithin‐PeQDs exhibit a longer average lifetime (*τ*_avg_ = 4.17 ns) and a reduced contribution from the fast decay component (*A*_1_). These findings clearly demonstrate that lecithin, as a capping ligand, effectively passivates surface defects, suppresses trap‐assisted nonradiative recombination, and thereby markedly improves the PLQY of PeQDs.

To gain deeper insight into the carrier dynamics, we performed transient absorption (TA) spectroscopy measurements. Pseudo‐color TA maps of the OAM‐PeQD film and the lecithin‐PeQD film under identical excitation energies are presented in Figure 3e,f, respectively, with their kinetics shown in Figure S20. A long‐lived photoinduced absorption (red) is observed above the bandgap (≈ 460 nm), which primarily originates from light‐induced changes in the imaginary part of the refractive index [73]. Concurrently, a pronounced negative absorption feature centered at ≈500 nm corresponds to ground‐state bleaching (GSB, blue), which is narrower for lecithin‐PeQDs, consistent with reduced band‐edge disorder and weaker defect‐related relaxation. To gain deep insight into the carrier dynamics in both PeQDs, the photobleaching temporal evolution from TA spectroscopy was normalized (Figure 3g) and fitted well with a biexponential function (Table S7). The fast component (*τ*_1_) originates from the presence of Auger recombination, while the slow component (*τ*_2_) is attributed to the intrinsic edge exciton recombination [74]. For OAM‐PeQDs, *τ*_1_ and *τ*_2_ (weight) are 5.1 ps (49%) and 47.1 ps (51%), respectively, and decrease to 4.2 ps (37%) and 42.2 ps (63%) for lecithin‐PeQDs with an increase in *τ*_avg_ to 33.4 ps from 20.4 ps. The decreased *τ*_1_ component and enlarged *τ*_avg_ undoubtedly indicate inhibition of Auger recombination by lecithin ligand, in line with TRPL results. Collectively, the experimental results indicate that lecithin can passivate the surface defects of PeQDs and suppresses the nonradiative Auger recombination process [75].

To further explore the ease of defect formation, we systematically investigated the thermal quenching behavior of both samples. The thermal quenching process primarily originates from the capture of charge carriers by thermally activated defects [76, 77]. Figure S21 presents pseudo‐color maps of the temperature‐dependent PL spectra for OAM‐PeQDs and lecithin‐PeQDs. As the temperature increases, the PL intensity of OAM‐PeQDs decreases sharply, retaining only 14.5% of their initial intensity at 373 K. In stark contrast, lecithin‐PeQDs maintain 27.0%, demonstrating improved thermal stability for lecithin‐PeQDs. To further quantify the energy barrier associated with defect formation, we fitted the temperature‐dependent PL data using the Arrhenius model (Equation 1):

(1) $$I_{PL}\;\left(T\right)=\frac{I_{PL}^{0}}{1+Ae^{-\frac{E_{a}}{k_{B}T}}}$$

where *I*_PL_(*T*) is the PL intensity at temperature *T*, $I_{\mathrm{PL}}^{0}$ is the initial intensity at 80 K, *k_B_* is the Boltzmann constant (8.617 × 10^−^^5^ eV/K), and *E_a_* is the activation energy [78]. The fitting results (Figure 3h) indicate that *E*_a_ for lecithin‐PeQDs is 57.2 meV, substantially higher than that of OAM‐PeQDs (37.8 meV). This higher *E*_a_ implies an increased energy barrier for defect formation in lecithin‐capped PeQDs, which aligns with their enhanced optical properties and suppressed Auger recombination [79].

To investigate the electron–phonon interaction, the temperature dependence of the PL linewidth (FWHM) was analyzed. The FWHMs of PL spectra for both samples broaden as temperature increases, arising from the recombination energy fluctuation due to the electron‐phonon coupling (Figure 3i). It should be pointed out that the broadening is less prominent for lecithin‐PeQDs, reflecting the weak exciton‐phonon coupling due to fewer surface defects [80]. The evolution of the PL linewidth was fitted using the Fröhlich coupling model (Figure 3i) [81] with the expression (Equation 2):

(2) $$\Gamma \left(T\right)=\Gamma_{0}+\gamma_{ac}T+\frac{\gamma_{LO}}{\exp\left(\frac{E_{LO}}{k_{B}T}\right)-1}$$

where Γ_0_ represents the temperature‐independent inhomogeneous broadening, γ_ac_ is the linear broadening coefficient originating from thermal expansion and acoustic‐phonon scattering, γ_*LO*_ denotes the exciton–longitudinal optical (LO) phonon coupling constant, *E_LO_* is the LO phonon energy, and *k_B_* is the Boltzmann constant. The fitting results reveal that lecithin‐PeQDs exhibit an electron–phonon coupling constant γ_LO_ of 25.8 meV, which is approximately 39.6% lower than that of OAM‐PeQDs (42.7 meV). Concurrently, *E*_LO_ decreases to 34.6 meV from 48.1 meV. The reduction in both γ_LO_ and *E*_LO_ provides direct evidence that lecithin modification effectively suppresses exciton–phonon interactions, which reduces non‐radiative recombination channels (Auger recombination) and thus improves PLQY, further validating the TRPL and TA spectroscopy [82]. Collectively, these characterizations reveal that lecithin ligand contributes to improving the defect energy formation and suppressing non‐radiative recombination channels relative by mitigating Auger recombination and electron–phonon interactions.

To further reveal the role of excitation density in Auger recombination, we performed pump‐fluence‐dependent TA measurements. The average exciton number per PeQD, ⟨N⟩, was estimated following the procedure detailed in the SI (see Eq.Equation S5), based on the measured pump photon fluence, film absorbance, film thickness, and number density of PeQDs. For both OAM‑PeQDs and lecithin‑PeQDs, the estimated ⟨N⟩ values with increasing pump fluence covered the transition from the near‑single‑exciton regime (⟨N⟩ < 1) to the multiexciton regime (⟨N⟩ > 1). As shown in Figure S22, under low excitation density (⟨N⟩ < 1) both samples exhibited weak fluence‑dependent acceleration of the bleaching recovery, consistent with dominant single‑exciton dynamics. When ⟨N⟩ exceeded unity, OAM‑PeQDs showed a pronounced acceleration of the early‑time bleaching recovery, indicating the activation of high‑density ultrafast nonradiative decay channels. In contrast, lecithin‑PeQDs exhibited a much weaker fluence‑dependent acceleration under comparable ⟨N⟩ values, with a relatively slower bleaching recovery (Figure S22c). This comparison supports that lecithin passivation suppresses multiexciton‑related ultrafast nonradiative recombination, including Auger‑assisted recombination.

To further corroborate the defect‐passivation effect of lecithin, we fabricated single‐carrier devices (hole‐only and electron‐only) and measured their current–voltage characteristics under dark conditions (Figure S23). The trap density was calculated using the trap‐filled limit voltage (*V*_TFL_) according to Equation (S6). As summarized in Table S8, the hole trap density of lecithin‐PeQDs is 1.7 × 10^1^ ^7^ cm^−^^3^, which is lower than that of OAM‐PeQDs (3.2 × 10^1^ ^7^ cm^−^^3^); similarly, the electron trap density decreases from 6.7 × 10^1^ ^7^ cm^−^^3^ (OAM‐PeQDs) to 3.8 × 10^1^ ^7^ cm^−^^3^ (lecithin‐PeQDs). These reduced trap densities directly confirm that lecithin passivation effectively decreases the density of defect‐related trap states, in good agreement with the optical evidence presented above.

### 2.4 Stability Evaluations

**FIGURE 4:**
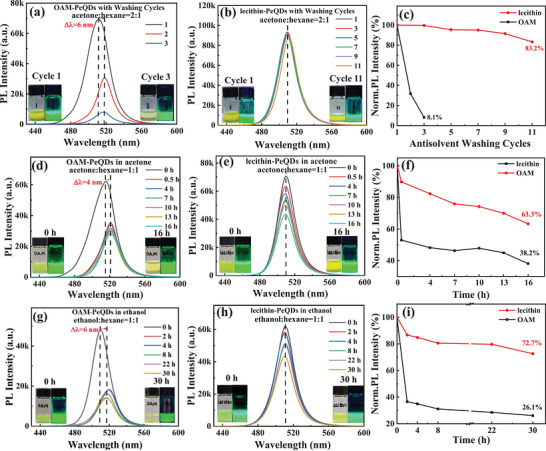
Stability assessment of PeQDs under polar conditions. PL spectral evolutions of (a) OAM‐PeQDs and (b) lecithin‐PeQDs in acetone/hexane (v:v = 2:1) mixture dispersion at 20 g/L after multiple washing cycles, and (c) their normalized PL intensities; PL spectral evolutions of (d) OAM‐PeQDs and (e) lecithin‐PeQDs in acetone/hexane (v:v = 1:1) mixture dispersion at 20 g/L and (f) their normalized PL intensities; PL spectral evolutions of (g) OAM‐PeQDs and (h) lecithin‐PeQDs in ethanol/hexane (v:v = 1:1) mixture dispersion at 20 g/L and (i) their normalized PL intensities.

The stabilities of both PeQDs against various polar solvents were first systematically evaluated. During anti‐solvent (acetone) purification, OAM‐PeQDs degrade severely after only 3 cycles, with their PL dropping to 8.1% of initial value and emission peak redshifting by ∼6 nm, as evidenced by the lighter green color and dimmer UV emission. In contrast, lecithin‐PeQDs maintain excellent stability after 11 cycles, retaining 83.2% of the original PL without any obvious spectral shift, as confirmed by their greenish transparency and bright UV emission in photographs (Figure 4a–c and Figure S24). In an acetone/hexane mixture (1:1), OAM‐PeQDs lose over 60% of PL intensity after 16 h, accompanied by a 4 nm redshift and visible precipitation. Under the same conditions, lecithin‐PeQDs retain 63.3% of their initial intensity with no observable peak shift (Figure 4d–f). A similar trend is observed in ethanol: after 30 h, OAM‐PeQDs exhibit a substantial decline to 26.1% along with a 6 nm redshift, whereas lecithin‐PeQDs maintain 72.7% of their intensity without shifting (Figure 4g–i). Water stability tests further confirm this trend (Figure S25). These results collectively demonstrate that lecithin, as a ligand, can significantly enhance the polar stability of PeQDs in polar solvents through the formation of a stable surface protective layer.

**FIGURE 5:**
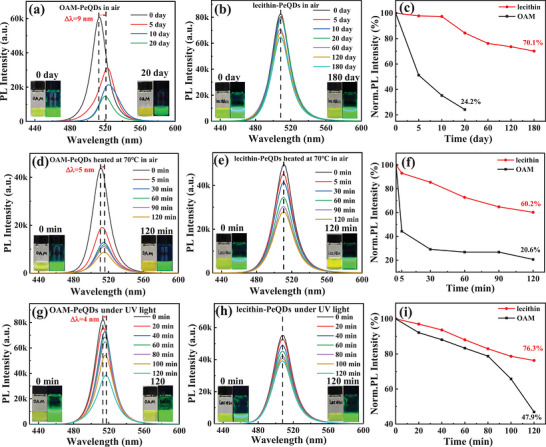
Stability assessment of PeQDs under various environmental conditions. PL spectral evolutions of (a) OAM‐PeQDs and (b) lecithin‐PeQDs in toluene dispersions at 20 g/L in air and (c) their normalized PL intensities. PL spectral evolutions of (d) OAM‐PeQDs and (e) lecithin‐PeQDs in toluene dispersions heated at 70°C for 120 min at 20 g/L and (f) their normalized PL intensities. PL spectral evolutions of (g) OAM‐PeQDs and (h) lecithin‐PeQDs in toluene dispersions at 20 g/L under 365 nm UV lamp irradiation and (i) their normalized PL intensities.

We further shift our attention to evaluate the environmental stabilities against storage, UV irradiation, and heat. At a concentration of 20 g/L in toluene, OAM‐PeQDs degrade significantly after 20‐day storage, with PL intensity dropping to 24.2% and a 9 nm redshift (Figure 5a). In stark contrast, lecithin‐PeQDs retain 70.1% of their initial PL intensity after 180 days with no peak shift, while the solution remains clear and fluorescent (Figure 5b). This enhanced stability applies to PeQDs at low concentration ∼0.5 g/L), where lecithin‐PeQDs maintain 82.2% of their PL intensity after 13 days vs. only 20.3% for OAM‐PeQDs (Figure S26). Thermal stability investigation at 70°C further highlights the superiority of the lecithin ligand. OAM‐PeQDs degrade rapidly, retaining only 20.6% of their initial PL intensity after 120 min and exhibiting a 5 nm redshift within the first 5 min, alongside increased turbidity and fluorescence quenching (Figure 5c,d). Conversely, lecithin‐PeQDs show excellent stability under the same conditions, maintaining 60.2% of their initial intensity with no significant peak shift, while the dispersion remains clear and highly fluorescent (Figure 5e). Their normalized PL intensities are shown in Figure 5f. We then evaluate their stability against UV irradiation. Figures 5g–i present the PL evolutions of the PeQDs in toluene dispersions (20 g/L) under irradiation of a 365 nm UV lamp (30 W) and the normalized fluorescence intensity. The results indicate that the decay rate of OAM‐PeQDs increases markedly after 120 min: their PL intensity retains only 47.9% of the initial value, accompanied by a red‐shift of the emission peak by about 4 nm. This result is consistent with the observed significant fluorescence quenching of the sample (inset of Figure 5g), suggesting that UV irradiation likely triggered ligand detachment and lattice damage. In contrast, lecithin‐PeQDs demonstrate excellent UV stability, with a significantly lower fluorescence decay rate compared to OAM‐PeQDs. After 120 min, they maintain 76.3% of their initial intensity without noticeable emission peak shift. The sample maintains good dispersibility and exhibits bright fluorescence (inset of Figure 5h), indicating that the zwitterionic protective layer formed by the lecithin ligands can effectively suppress the UV‐induced degradation process, thereby significantly enhancing the photostability and structural integrity of the PeQDs. In total, the lecithin ligand enables PeQDs to exhibit prominent resistance to polar solvents and external stimuli.

To further evaluate the practical applicability of PeQDs, especially considering that PeQDs are typically fabricated into films for solid‐state optoelectronic devices, we systematically investigated the stability of OAM‐PeQDs and lecithin‐PeQDs in the film state, including thermal stability, UV‐irradiation stability, and ambient storage stability (Figures S27–S29). For thermal stability, the films were heated in air at 70°C for 60 min. As shown in Figure S27, the PL intensity of the OAM‐PeQD film decreased to 36.8% of its initial value after heating, whereas the lecithin‐PeQD film retained 67.7% of its initial PL intensity under the same conditions. This indicates that lecithin passivation effectively improves the thermal robustness of PeQD films. We further evaluated the film stability under continuous 365 nm UV irradiation. As shown in Figure S28, the OAM‐PeQD film exhibited obvious yellowing after 3 days and became nearly non‐emissive after 9 days. In contrast, the lecithin‐PeQD film still showed bright green emission after 9 days of UV exposure, suggesting substantially enhanced photostability in the film state. In addition, the films were stored in ambient air without encapsulation to assess their storage stability. After 20 days, the OAM‐PeQD film became almost non‐emissive, whereas the lecithin‐PeQD film still maintained strong PL, as shown in Figure S29. This result further supports the improved storage stability in air of lecithin‐PeQD films. Overall, these film‐state measurements demonstrate that lecithin‐PeQD films exhibit improved thermal, UV‐irradiation, and ambient storage stability compared with OAM‐PeQD films.

We further tested the humidity resistance of lecithin‑PeQDs under ambient conditions. Lecithin‑PeQD films and solutions were stored in a low‑humidity glovebox and under ca. 80% relative humidity (RH). The lecithin‐PeQDs maintained bright emission under both conditions (Figures S30 and S31), and after 14 days the PL intensity of the solutions remained 82.0% (glovebox) and 70.9% (ca. 80% RH) of their initial values. These results confirm that lecithin‑PeQDs possess good humidity resistance, supporting the feasibility of the proposed large‑scale synthesis under ambient conditions.

### 2.5 ASE Performance

**FIGURE 6:**
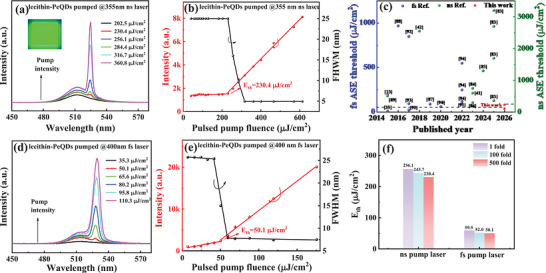
ASE performances of lecithin‐PeQDs films. Emission spectra of the lecithin‐PeQDs film under (a) ns and (d) fs pulsed laser excitation at different pump energies. ASE threshold and FWHM evolution of the lecithin‐PeQDs film under (b) ns and (e) fs pulsed laser excitation. (c) Summary of reported optical gain thresholds for PeQDs from literature under ns and fs pulsed laser excitation. (f) ASE threshold of lecithin‑PeQDs films as a function of reaction fold (1, 100, 500) under ns and fs pulsed laser excitation.

With the excellent optical properties and colloidal stabilities in mind, it is expected for lecithin‐PeQDs to serve as a promising gain medium in the ASE field. To validate this point, thin films from both samples were fabricated via direct spin‐coating, and their ASE performances were systematically investigated. The emission spectra of both films under different pump energy excitations by an ns laser are shown in Figure 6a and Figure S32. For the OAM‐PeQD film, only PL emission was observed across all excitations, with no occurrence of ASE. In contrast, the lecithin‐PeQD film exhibits PL emission at low pump flux with a FWHM of 25 nm. As the pump flux surpasses the threshold, the output intensity rises sharply, accompanied by the emergence of a narrowband peak centered at approximately 525 nm and a significant reduction of FWHM to ∼ 5 nm, indicating the onset of ASE. The ASE threshold and FWHM evolution under ns excitation are presented in Figure 6b. The dependence of output emission intensity and FWHM on pump flux was employed to determine the threshold (Figure 6c), where a clear knee point can be easily found. As a result, the ASE threshold is determined to be 230.4 µJ cm^−2^, which is notably lower than reported values for neat CsPbBr_3_ PeQDs under comparable ns‐pulsed excitation (Figure 6c and Table S9) [23, 41, 42, 83, 84, 85, 86], confirming the excellent ASE performance of the lecithin‐PeQDs. Meanwhile, ASE performance was also investigated by using fs laser pumping. Similarly, the OAM‐PeQDs only present PL emission during the whole pump flux (Figure S26b), while the lecithin‐PeQD film exhibits typical ASE characteristics, with a threshold of 50.1 µJ cm^−2^ (Figure 6d,e). This is much smaller than that by ns pumping and is comparable to many reported values for neat PeQD‐based ASE using an fs laser as a pump (Figure 6c and Table S10) [23, 87, 88, 89, 90, 91, 92, 93, 94]. demonstrating clear ASE behavior with good operational stability.

In addition to the intrinsic optical properties of PeQDs, film morphology and surface roughness can also influence ASE behavior by affecting optical scattering, waveguiding, and local gain uniformity. To examine this aspect, we performed atomic force microscopy (AFM) and scanning electron microscopy (SEM) measurements on the OAM‑PeQD and lecithin‑PeQD films (Figures S33 and S34). The root‑mean‑square (RMS) roughness values are 6.5 nm for the OAM‑PeQD film and 5.4 nm for the lecithin‑PeQD film (Figure S33). The lecithin‑PeQD film thus exhibits a smoother and more uniform surface morphology, as also supported by the SEM images (Figure S34). Such morphological differences can reduce optical scattering losses and improve waveguiding efficiency, thereby potentially lowering the ASE threshold. Therefore, the improved ASE characteristics of lecithin‑PeQD films are likely associated not only with enhanced surface passivation and optical quality, but also with the favorable film morphology.

To clarify whether batch‑prepared lecithin‑PeQDs exhibit ASE and whether the ASE characteristics vary with preparation scale, we investigated the ASE behavior of a 500‑fold lecithin‑PeQD film under both ns and fs pulsed laser excitation. For comparison, the ASE properties of 1‑fold and 100‑fold films are provided in Figures S35 and S36. Under ns pulsed excitation, the 500‑fold film showed typical ASE behavior, characterized by a rapid increase in emission intensity and pronounced spectral narrowing above the threshold. The ASE threshold was determined to be 238.5 µJ cm^−^^2^ for the 500‑fold film (see Figure 6f). This value is comparable to those of the 1‑fold and 100‑fold films (256.1 and 243.7 µJ cm^−^^2^, respectively, as shown in Figures S35b,d), indicating that scale‑up preparation does not significantly degrade the ASE performance, even up to 500‑fold. Similarly, under fs pulsed excitation, clear ASE was observed for the 500‑fold film. The ASE threshold was 50.1 µJ cm^−^^2^ (Figure 6f), which is consistent with the thresholds of the 1‑fold (59.5 µJ cm^−^^2^) and 100‑fold (52.0 µJ cm^−^^2^) samples (Figure S36b,d). The consistent thresholds and spectral evolution further confirm that the ASE properties of lecithin‐PeQDs are well preserved after scale‐up. Taken together, these results demonstrate that batch‐prepared lecithin‐PeQDs retain reproducible ASE characteristics across different preparation scales (1‐fold, 100‐fold and 500‐fold), supporting the scalability of the lecithin‐assisted synthesis strategy.

It is well known that net gain is a critical parameter to evaluate optical amplification capability. The gain of the thin film was measured and fitted using the variable stripe length (VSL) method. Excitation light is shaped into a stripe‐shaped spot using a cylindrical lens, while emission intensity is measured as a function of the excited stripe length *L*. The acquired data is then fitted with the equation of (Equation 3):

(3) $$I=\frac{A\left(\lambda \right)I_{p}}{G\left(\lambda \right)}\left(e^{G\left(\lambda \right)L}-1\right)$$

where *A*(λ) is the spontaneous emission factor, *I_p_* denotes the pump intensity, *G*(λ) represents the net gain coefficient, and *L* is the excitation stripe length. We measured the net gain coefficients of the lecithin‐PeQD film over an extended stripe length range of 0–1 mm, carefully avoiding the film edge to minimize artifacts from thickness inhomogeneities. Under both ns and fs excitation, the excitation density was fixed at 400 µJ cm^−^^2^ to ensure comparable carrier injection conditions. The extracted gain coefficients are 48.5 ± 4.1 cm^−^^1^ under ns excitation and 287.1 ± 7.2 cm^−^^1^ under fs excitation (Figures S37 and S38). These values indicate a substantial optical amplification capability of the film. We further evaluated the ASE stability of the lecithin‐PeQDs films. As shown in Figure S39, under ns laser pulse excitation for 120 min, the ASE intensity remained 100% of its initial value; under fs laser pulse excitation for 60 min, it retained 81.5% of its original intensity. These results demonstrate that the lecithin‐PeQD films possess excellent ASE stability.

## 3 Conclusions

In summary, we have developed a room‐temperature green synthesis strategy utilizing bio‐sourced, zwitterionic lecithin as a ligand to enable the scalable production of high‐quality CsPbX_3_ PeQDs. Theoretical calculations reveal that lecithin passivation follows a unique bidentate synergistic mechanism: its negatively charged phosphate group covalently coordinates with undercoordinated Pb^2^^+^, while the positively charged choline group electrostatically interacts with surface Br^−^ ions. This dual binding mode effectively suppresses Ostwald ripening, as corroborated by in‐situ PL, FTIR, and XPS analysis. Leveraging this stability, the reaction was successfully scaled up by 500‐fold with a prolonged duration of 120 min, yielding over 10 grams of PeQDs per batch without compromising their optical properties, morphological uniformity, or crystal structure. Systematic characterizations demonstrate that lecithin efficiently passivates surface defects, significantly suppressing non‐radiative recombination channels from both Auger processes and electron‐phonon coupling. The resulting PeQDs also exhibit markedly enhanced stability under various harsh conditions, including ambient exposure, heat, UV irradiation, and polar solvents. These superior attributes collectively enable state‐of‐the‐art ASE performance with low thresholds and high net gain coefficients under both nanosecond and femtosecond laser excitation. This work not only provides an efficient, green, and scalable route to highly stable and efficient PeQDs, but also opens a new avenue for zwitterionic ligand design, advancing the practical application of PeQDs in high‐performance optoelectronic devices such as nanolasers.

## Author Contributions

**Mengting Zhang**: data curation, formal analysis, software, Writing – original draft, visualization. **Jia Wang**: data curation, software, resources. **Qingyu Xie**: software, validation. **Min Zhou**: writing – review and editing, methodology, software. **Juan Du**: data curation, conceptualization. **Yongfeng Liu**: conceptualization, methodology, investigation, formal analysis, supervision, resources, project administration, writing – review and editing, funding acquisition. **Bowen Zhang**: software. **Jie Yang**: data curation. **Kehao Hu**: data curation. **Zhe Zhang**: software. **Zhiping Hu**: data curation.

## Conflicts of Interest

The authors declare no conflicts of interest.

## Supporting information




**Supporting File**: advs76783‐sup‐0001‐SuppMat.docx.

## Data Availability

The data that support the findings of this study are available on request from the corresponding author. The data are not publicly available due to privacy or ethical restrictions.
