# Supplementary material for: Green and Scalable Synthesis of Efficient and Stable CsPbBr3 Perovskite Quantum Dots Enables Low‐Threshold Amplified Spontaneous Emission
Source: Adv Sci (Weinh). 2026 Jul 27;13(58):e76783. doi: 10.1002/advs.76783 (PMC13403731; doi:10.1002/advs.76783)
Supplement: Supplementary file 1 [file ADVS-13-e76783-s001.docx]

**Supporting Information for**

**Green and scalable synthesis of efficient and stable CsPbBr_3_ perovskite quantum dots enables low-threshold amplified spontaneous emission**

Yongfeng Liu^a,*, #^, Mengting Zhang^a,#^, Qingyu Xie^a,#^, Kehao Hu ^b,#^, Bowen Zhang^a^, Zhe Zhang^a^, Jie Yang^c,*^, Zhiping Hu^b^, Juan Du^b,*^,Jia Wang^d,e,*^, Min Zhou ^a,*^

^a^ College of Physical Science and Technology, Yangzhou University, Yangzhou 225002, China

^b^ School of Physics and Optoelectronic Engineering, Hangzhou Institute for Advanced Study, University of Chinese Academy of Sciences, Hangzhou 310024, Zhejiang, China

^c^ College of Physics and Optoelectronic Engineering, Chongqing Normal University, Chongqing 401331, China

^d^ Department of Physics, Umeå University, Umeå SE-90187, Sweden

^e^ Wallenberg Initiative Materials Science for Sustainability, Department of Physics, Umeå University, SE-90187 Umeå Sweden.

*Corresponding authors. E-mail address: liuyf@yzu.edu.cn, jieyang611@cqnu.edu.cn, du@ucas.ac.cn, jia.wang@umu.se, and minzhou@yzu.edu.cn.

^#^These authors contribute equally to this work.

**1. Experiments**

**1.1 Chemicals**

Cesium carbonate (Cs_2_CO_3_, 99.9%), lecithin (98%), oleylamine (OAM, 80-90%), n-hexane (97%) were purchased from Macklin. Lead acetate trihydrate (Pb(Ac)_2_·3H_2_O, 99.99%), tetraoctylammonium bromide (TOAB, 98%), n-octanoic acid (OTAc, ≥99%) were purchased from Aladdin. Toluene (≥98%), acetone (99.5%) were purchased from Shanghai Lingfeng chemical reagent Co., Ltd. All these reagents were used without further purification.

**1.2 Preparation of Precursors**

In order to obtain the precursor solutions of Cs-OTAc and Pb-OTAc, Cs_2_CO_3_ (15 mmol) and Pb(Ac)_2_·3H_2_O (30 mmol) were dissolved at room temperature in OTAc (200 mL), respectively. For the Br-OTAc-hexane solution, TOAB (18 mmol) was dissolved in OTAc (200 mL) and hexane (400 mL). The lecithin-OTAc precursor solution was prepared by completely dissolving lecithin (9 mmol) in OTAc (150 mL) at room temperature. The TOAB (18 mmol) was dissolved in a mixed solvent system containing OTAc (200 mL) and toluene (400 mL) under magnetic stirring, yielding the homogeneous Br-OTAc-toluene precursor solution. The OAM precursor solution was prepared through complete dissolution of OAM (30 mmol) in OTAc (200 mL) and toluene (500 mL) under continuous stirring at room temperature.

**1.3 Synthesis and purification**

**Synthesis of lecithin-PeQDs:** The Pb-OTAc precursor solution (0.25 mL) was thoroughly mixed with hexane (1 mL) at room temperature until complete dissolution, yielding a homogeneous Pb-OTAc-hexane solution (1.25 mL). The Cs-OTAc (0.15 mL), Pb-OTAc-hexane (1.25 mL) and Br-OTAc-hexane precursor solution (4.5 mL) were combined in a 50 mL conical flask. The mixture was magnetically stirred for 10 min to ensure complete dissolution. Then, lecithin-OTAc precursor solution (1.5 mL) was rapidly injected into the above mixture under vigorous stirring. The reaction was allowed to proceed for 6 min at ambient conditions. The crude solution was immediately mixed with twice its volume of acetone to induce PeQD precipitation. Centrifugation was performed at 10000 rpm for 2 min to collect the precipitate. The pellet was redispersed in hexane (2 mL). A second centrifugation at 8000 rpm for 2 min effectively removed larger aggregates, with the supernatant containing purified lecithin-capped PeQDs being collected for subsequent characterization. The same procedure could be directly scaled up by 500 times for bulk production.

**Synthesis of OAM-PeQDs**: The Cs-OTAc (0.5 mL), Pb-OTAc (1 mL), and OAM-OTAc-toluene precursor solution (4.5 mL) were stirred in a 50 mL conical flask until completely dissolved. Subsequently, the Br-OTAc-toluene precursor solution (0.5 mL) was rapidly injected into the mixture, and the reaction was allowed to proceed for 2 min. The resulting crude solution was mixed with twice the volume of ethyl acetate and centrifuged at 10000 rpm for 2 min to collect the precipitate. The precipitate was then redispersed in 2 mL of toluene and centrifuged at 8000 rpm for 2 min. Finally, the supernatant was collected to obtain OAM-PeQDs.

**Synthesis of CsPbX_3_ (X=I and Cl) PeQDs:** A lecithin-PeQD dispersion in n-hexane was treated with pre-synthesized zinc halide solution. The zinc halide solution was prepared by dissolving zinc halide (ZnX_2_, X=I or Cl) (0.03 mmol) in a mixture of n-hexane (2 mL) and OAM (100 μL) under ambient temperature conditions. Subsequently, the predetermined amount of zinc halide solution was introduced into 1 mL of lecithin-CsPbBr_3_ PeQD n-hexane solution (approximately 0.1 g/L) under constant stirring.

**1.4 Preparation of PeQD films and ASE** **measurements**

A 30 g/L n-hexane solution of CsPbBr_3_-PeQDs was spin-coated onto glass substrates at 1000 rpm for 60 s under ambient conditions to form highly smooth thin films. Nanosecond pulses were delivered at 355 nm by a solid-state Nd:YAG laser (minite Ⅱ Q-switched Nd:YAG) operating at 10 Hz repetition rate, with pulse duration of 3-7 ns. The output pulse energy of the pump beam was controlled using neutral density filters. Femtosecond pulses were delivered at 400 nm by a Ti:Sapphire amplifier system operating at a 1 kHz repetition rate, with a pulse duration of 50 fs. The pump intensity was measured with a calibrated laser power and energy meter (Gentec). The beam was focused using a cylindrical lens to form a narrow stripe, and the excitation light was vertically incident on the sample. Emitted light was collimated via an optical fiber and coupled into an imaging spectroradiometer (Horiba, iHR-320). The optical gain of the sample was measured at room temperature under ambient conditions using the standard VSL method. The excitation stripe length was varied by a micrometer-driven adjustable slit positioned at the focal line of the cylindrical lens.

**1.5** **Characterizations**

Absorption spectra we recorded using a UV–vis spectrophotometer (Cary 60, Agilent Technologies). FTIR spectra were obtained with an FTIR spectrometer (670-IR, Varian). PL and PLQY were measured using a custom-built system incorporating an integrating sphere and a spectrometer (QEPRO, Ocean Insight). TA was performed on a femtosecond transient absorption spectrometer (TA100, Dalian Chuangrui Optics). TEM images were acquired using a thermal field emission transmission electron microscope (G2 F30 S-TWIN, Tecnai). TGA was carried out on a TGA 5500 instrument (TA Instruments) under a nitrogen atmosphere (flow rate: 40 mL/min) with a heating rate of 10 ℃/min. TRPL decay curves were measured using a fluorescence lifetime spectrometer (FLS980, Edinburgh Instruments). XPS analysis was conducted on an XPS spectrometer (ESCALAB 250Xi, Thermo Fisher Scientific). XRD patterns were recorded on a Shimadzu XRD-7000 diffractometer using Cu Kα radiation (λ = 1.5405 Å).

**1.6 Calculation**

First-principles calculations were performed within the framework of spin-polarized DFT implemented in the Vienna Ab initio Simulation Package (VASP). The exchange-correlation interactions and electron-ion interactions were described using the Perdew-Burke-Ernzerhof (PBE) generalized gradient approximation functional and the projector augmented wave (PAW) method, respectively. The DFT-D3 method was employed to account for long-range van der Waals interactions. In all calculations, the plane-wave cutoff energy was set to 450 eV, with convergence thresholds of 10^-5^ eV for energy and 0.02 eV Å^-1^ for forces. To mitigate periodic boundary effects, a 2×3×1 supercell was adopted with a 20-Å vacuum layer along the z-direction. Geometric optimizations utilized a 3×3×1 k-point mesh sampling over the Brillouin zone. The binding energy (E_b_) between CsPbBr_3_ PeQDs and OAM/lecithin ligand was calculated as:

$$\begin{aligned} E_{b}= E_{total}- E_{ligand}- E_{PeQDs}\#\left（ S1 \right） \end{aligned}$$

where $E_{total}$denotes the total energy of the ligand-adsorbed PeQD system, while $E_{ligand}$ and $E_{PeQDs}$ represent the energies of the isolated ligand and pristine PeQD, respectively.

**Fig. S1.** Chemical structures of lecithin, OAM and OTAc.

**Fig. S2.** Chemical structures of the simplified OAM model and the simplified lecithin model.

**Fig. S3.** DOS of CsPbBr_3_ PeQDs capped with (a) the simplified OAM model and (b) the simplified lecithin model.

**Fig. S4.** Lecithin-PeQDs synthesized under different conditions: (a) PL spectra with various acids, (b) PL spectra with different precursor mmol amounts, (c) PL spectra with varying Cs:Pb:Br ratios, and (d) corresponding PLQY bar chart comparison.

**Fig. S5.** OAM-PeQDs synthesized under different conditions: (a) PL spectra with various OAM volumes, (b) PL spectra with varying Cs:Pb:Br ratios, and (c) corresponding PLQY bar chart comparison.

**Fig. S6.** (a) PL spectrum of OAM-PeQDs in hexane; (b) UV–vis absorption of OAM-PeQDs in hexane.

**Fig. S7.** (a) PL spectra, (b) UV–vis absorption spectra, and (c) PLQY bar chart of OAM-PeQDs at different reaction scaling factors**.**

**Fig. S8.** (a) TEM image and (b) size distribution of OAM-PeQDs at the 10-fold scale.

The reported size was calculated as the average of the measured length and width for each nanocrystal.

**Fig. S9.** (a) Daylight and (b) UV-light fluorescence images of OAM-PeQDs at different reaction scaling factors.

**Fig. S10.** (a) PL spectra, (b) UV–vis absorption spectra, and (c) PLQY bar chart of lecithin-PeQDs at different reaction scaling factors**.**

**Table S1.** Summary of Batch Synthesis Strategies and Properties for CsPbBr₃ PeQDs via wet chemistry.

| Ligands | Synthesis method | Reaction  span | PLQY | production | Production  Yield | Ref. |
| --- | --- | --- | --- | --- | --- | --- |
| ASC14 | HI | 120 min | 87% | 0.2 g | NG | ^[58]^ |
| ASC18 | HI | 0.1 min | 95% | 0.3 g | NG | ^[60]^ |
| ASC18 | HI | Instant | 87% | 1.1 g | NG | ^[59]^ |
| OA | LARP | NG | 80% | ~1 g | NG | ^[9]^ |
| ASC18 | LARP | 120 min | 92% | 1.8 g | 68.8% | ^[21]^ |
| MS-NB | HI | 3.3 min | ~100% | 6 g | NG | ^[57]^ |
| OA, OAM | LARP | NG | 80% | ~1 g | 60% | ^[55]^ |
| OA, OAM | microwave-assisted | 5 min | 94% | 5 g | NG | ^[56]^ |
| OA, OAM | One-pot | 20 min | 77% | ~1 g | NG | ^[54]^ |
| OTAc, lecithin | LARP | 120 min | 95% | 10.1 g | 81.4% | This work |

Notes

Production: The mass of purified PeQDs obtained after the complete synthesis, washing, and isolation process from a single batch. It is typically reported in grams (g) or milligrams (mg).

**Production yield:** The ratio of the actual production (mass of purified PeQDs) to the total mass of all precursor materials used in the synthesis. It is expressed as a percentage (%).

**Table S2.** Weight statistics of the PeQD samples for yield calculations.

| Sample | lecithin-500-fold | OAM-5-fold |
| --- | --- | --- |
| yield (mg) | 10046.1 | 41.3 |

The theoretical weight of 0.0375 mmol CsPbBr_3_ is about 21.7 mg, and the yield is calculated as follows:

$${yield}_{lecithin}=\frac{m_{puried}}{n\cdot M}=\frac{20.09\times89.3\%}{21.7425}=82.5\%$$

$${yield}_{OAM}=\frac{m_{puried}}{n\cdot M}=\frac{8.26\times84.9\%}{21.7425}=32.4\%$$

where $m_{purified}$ is the mass of the dried quantum dots, $\omega\%$ is the mass percentage of CsPbBr_3_ (deduced from the results of thermogravimetric analysis).

**Fig. S11.** (a) PL spectra under 365 nm excitation, (b) UV–vis absorption, (c) FTIR and (d) XRD patterns of lecithin-PeQDs at different reaction times.

**Fig. S12.** TEM images of lecithin-PeQDs at different reaction times: (a) 30 min, (b) 60 min, (c) 90 min, (d) 120 min, with corresponding size distribution histograms.

**Fig. S13.** High-resolution XPS in the P 2p region.

**Fig. S14.** XPS survey spectra of the OAM-PeQDs and lecithin-PeQDs powder and their corresponding element ratios.

**Table S3.** The elemental ratios obtained from XPS measurements.

| Sample | Cs3d5 | Pb4f | Br3d | C1s | O1s | N1s | P2p |
| --- | --- | --- | --- | --- | --- | --- | --- |
| OAM | 1.1 | 1.0 | 3.4 | 35.2 | 2.4 | 1.5 | \ |
| lecithin | 1.8 | 1.0 | 3.6 | 19.1 | 52.6 | 3.1 | 2.7 |

**Table S4.** Summary of Cs:Pb:Br atomic ratios for CsPbBr_3_ PeQDs from XPS analysis.

| Cs:Pb:Br | Ref. |
| --- | --- |
| 1:1:3.4 | ^[1]^ |
| 1.5:1:3.9 | ^[2]^ |
| 1.1:1:3.5 | ^[3]^ |
| 1.1:1:3.4 and 1.8:1:3.6 | This work |

**Fig. S1****5**. Size distribution of OAM and lecithin-PeQDs.

**Fig. S16.** TGA of OAM-PeQDs and lecithin-PeQDs.

**Equation for calculation of capping ligand density:**

$\text{σ}\text{=}\frac{\text{M}_{\text{total}}\text{*}{\text{ω}\text{t\%}}_{\text{ligand}}\text{*}\text{N}_{\text{A}}}{\text{M}_{\text{w}}}\text{/(}\frac{\text{M}_{\text{total}}\text{*}{\text{ω}\text{t\%}}_{\text{core}}}{\text{ρ}_{\text{core}}\text{*}\text{L}_{\text{core}}^{\text{3}}}\text{*6}\text{L}_{\text{core}}^{\text{2}}\text{)}$	(*S*2)

where σ is the capping ligand density sitting on the PeQD core; $M_{total}$ is the PeQDs mass including capping ligand and core (Note: any value as it will be cancelled out from the numerator and the denominator); ${\omega t\%}_{ligand}$ is the mass fraction of organic capping ligand compared to the entire PeQDs, which obtained from TGA measurement; ωt %_core_ is the mass fraction of inorganic core compared to the entire PeQDs; $M_{w}$ is the molecular weight of the ligand lecithin (758.1 g/mol) or OTAc (144.2 g/mol) ; NA is Avogadro’s constant; $L_{core}$ is the average edge length of the PeQDs, obtained from TEM; ρ_core_ is the density of the PeQDs core (4.8 g/cm3), taken from the reference; The obtained results are shown in Table S4.

**Table S5.** Summary of the calculated capping ligand densities based on TGA, XPS and TEM.

| PeQDs | Mass fraction of ligand (%) | | σ (/nm^2^) | | |
| --- | --- | --- | --- | --- | --- |
|  | OTAc | OAM/lecithin | OTAc | OAM/lecithin | total |
| OAM-PeQDs | 3.4 | 11.7 | 1.7 | 3.1 | 4.8 |
| lecithin-PeQDs | 4.0 | 6.7 | 1.4 | 0.8 | 2.3 |

**Fig. S17.** PLQY of OAM-PeQDs and lecithin-PeQDs in the film state.

**Fig. S18.** Photograph of CsPbX_3_ PeQDs dispersions under UV light.

**Fig. S19.** (a) PL spectra of CsPbX_3_ PeQDs dispersions. (b) Normalized PL intensity as a function of time.

**Equations for lifetime calculation:**

$$\begin{aligned} I\left( t \right)= I_{0}+A_{1}\exp\left( -\frac{t}{\tau_{1}} \right)+A_{2}\exp\left( -\frac{t}{\tau_{2}} \right)\#\left( S3 \right) \end{aligned}$$

$$\begin{aligned} \tau_{avg}=\frac{A_{1}*\tau_{1}^{2}+A_{2}*\tau_{2}^{2}}{A_{1}*\tau_{1}+A_{2}*\tau_{2}}\#\left( S4 \right) \end{aligned}$$

**Table S6.** Fitted lifetime parameters of PeQDs.

| PeQDs | ***A***_1_ | **τ**_1_ (ns) | ***A***_2_ | **τ**_2_ (ns) | **τ**_avg_ (ns) |
| --- | --- | --- | --- | --- | --- |
| OAM | 0.66 | 0.86 | 0.32 | 4.39 | 3.37 |
| lecithin | 0.71 | 0.98 | 0.24 | 5.68 | 4.17 |

**Fig. S20.** GSB kinetics extracted from the time-resolved TA spectra of (a) OAM-PeQDs and (b) lecithin-PeQDs.

**Table S7.** Summary of TA spectra fitting data for OAM-PeQDs and lecithin-PeQDs.

| PeQDs | ***A***_1_ | ***τ***_1_(ps) | ***A***_2_ | ***τ***_2_(ps) | ***τ****_avg_*(ps) |
| --- | --- | --- | --- | --- | --- |
| OAM-PeQDs | 0.4 | 5.1 | 0.4 | 47.1 | 20.4 |
| lecithin-PeQDs | 0.3 | 4.2 | 0.5 | 42.2 | 34.0 |

**Fig. S21.** Temperature-dependent emission spectra of (a) OAM and (b) lecithin-PeQDs.

**Fig. S22.** Pump-fluence-dependent transient absorption analysis of OAM-PeQDs and lecithin-PeQDs. (a, b) Bleaching recovery kinetics of OAM-PeQDs and lecithin-PeQDs extracted at the GSB maximum under different estimated average exciton numbers ⟨N⟩. (c) Normalized bleaching recovery kinetics of OAM-PeQDs and lecithin-PeQDs at high excitation density, showing a faster early-time recovery for OAM-PeQDs than for lecithin-PeQDs.

The average exciton number per PeQD, <N>, is calculated as:

$$\begin{aligned} <N> = \frac{J\cdot\left( 1-{10}^{-A_{film}} \right)}{n\cdot d}\#\left( S5 \right) \end{aligned}$$

where *J* is the pump photon fluence, *A_film_* is the film absorbance at the pump wavelength, *n* is the number density of PeQDs in the film, and *d* is the film thickness.

**Fig. S23.** Current density–voltage curves of the (a) hole-only device and (b) electron-only device under dark conditions. The inset shows the scheme of the device structure.

The trap density η_trap_ is linearly related to the trap-filled limit voltage (𝑉𝑇𝐹𝐿):

$$\begin{aligned} \eta_{trap}=\frac{2\varepsilon\varepsilon_{0}V_{TFL}}{eL^{2}}\#\left( S6 \right) \end{aligned}$$

where $\varepsilon_{0}$ is the vacuum dielectric constant, $\varepsilon$ is the relative dielectric constant of CsPbBr_3_ (𝜀 = 4.32), $L$ is the thickness of CsPbBr_3_ perovskite film. The hole trap densities for the lecithin-PeQD and OAM-PeQD films were calculated to be 1.7×10^17^ and 3.2×10^17^ cm^−3^, respectively. The electron trap density was reduced from 6.7×10^17^ to 3.8×10^17^ cm^−3^ after changing the ligands to lecithin.

**Table S8.** Trap densities of OAM-PeQDs and lecithin-PeQDs from single-carrier devices.

| PeQDs | Hole trap density (cm^-3^) | Electron trap density (cm^-3^) |
| --- | --- | --- |
| OAM-PeQDs | 3.2×10^17^ | 6.7×10^17^ |
| lecithin-PeQDs | 1.7×10^17^ | 3.8×10^17^ |

**Fig. S24.** Photographs of (a) OAM-PeQDs and (b) lecithin-PeQDs in daylight and under 365 nm UV lamp irradiation after muiliple antisolvent washing cycles.

**Fig. S25.** (a) PL spectral evolutions of (a) OAM-PeQDs and (b) lecithin-PeQDs dispersions in hexane/water (v:v=10:1) mixtures at 3 g/L and (c) their normalized PL intensities.

**Fig. S26.** PL spectral evolutions of (a) OAM-PeQDs and (b) lecithin-PeQDs in toluene dispersions at 0.5 g/L and (c) their normalized PL intensities.

**Fig. S27.** PL spectral evolutions of (d) OAM-PeQDs film and (e) lecithin-PeQDs film heated at 70 °C for 60 min, and (f) their normalized PL intensities.

**Fig. S28.** Stability of lecithin-PeQDs and OAM-PeQDs films under UV lamp illumination: (a) under a daylight lamp; (b) under a UV lamp.

**Fig. S29.** Storage stability of lecithin-PeQDs and OAM-PeQDs films: (a) under a daylight lamp; (b) under a UV lamp.

**Fig. S30.** PL spectral evolutions of lecithin-PeQDs solutions (a) in a glovebox, (b) under ~80% relative humidity over time, and (c) their normalized PL intensities under the two humidity conditions.

**Fig. S31.** Photographs of lecithin-PeQDs films under different humidity conditions: (a) under a daylight lamp; (b) under a UV lamp. The films were stored in a glovebox and under ~80% relative humidity.

**Fig. S32.** Emission spectra of the OAM-PeQDs film under (a) ns and (b) fs pulsed laser excitation at different pump energies.

**Fig. S33.**AFM images of (a) lecithin‑PeQDs and (b) OAM‑PeQDs.

**Fig. S34.** SEM images of (a) lecithin‑PeQDs and (b) OAM‑PeQDs.

**Fig. S35.** Emission spectra of (a) 1-fold lecithin-PeQD film and (c) 100-fold lecithin-PeQD film under ns pulsed laser excitation at different pump energies. ASE threshold and FWHM evolution of (b) 1-fold lecithin-PeQDs and (d) 100-fold lecithin-PeQDs under ns pulsed laser excitation.

**Fig. S36.** Emission spectra of (a) 1-fold lecithin-PeQD film and (c) 100-fold lecithin-PeQD film under fs pulsed laser excitation at different pump energies. ASE threshold and FWHM evolution of (b) 1-fold lecithin-PeQDs and (d) 100-fold lecithin-PeQDs under fs pulsed laser excitation.

**Table S9.** Optical gain performance parameters of CsPbBr_3_ PeQDs spin-coating films under ns pulsed pumping.

| Ligand | Pulse source | Threshold (μJ/cm^2^) | Gain (cm^-1^) | Ref. |
| --- | --- | --- | --- | --- |
| lecithin | 337 nm/3 ns | 600 | NG | _[41]_ |
| OAM and OA | 337 nm/3 ns | 2550 | 12.9 | _[42]_ |
| OAM and OA | 337 nm/3 ns | 2700 | NG | _[83]_ |
| DDAB |  | 1700 | NG |  |
| ASC18 |  | 3200 | NG |  |
| lecithin |  | 360 | NG |  |
| ASC18 and OA | 337 nm/3 ns | 750 | 23.1±0.8 | _[84]_ |
| DDAB | 337 nm/3 ns | 1300 | 23.0±2.1 | _[85]_ |
|  | 355 nm/10 ns | 450±50 | NG | _[23]_ |
| OAM and OA | 532 nm/10 ns | 300 | NG | _[86]_ |
| lecithin | 355 nm/3-7 ns | 230.4 | 48.5±4.1 | This work |

Notes

ACS18: N,N-dimethyloleylammonio)propanesulfonate

DDAB: dimethyldidodecylammonium bromide

**Table S10.** Optical gain performance parameters of CsPbBr_3_ PeQDs spin-coating films under fs pulsed pumping.

| Ligands | Pulse source | Threshold (μJ/cm^2^) | Gain (cm^-1^) | Ref. |
| --- | --- | --- | --- | --- |
| 2-hexyldecanoic acid and OAM | 400 nm/50 fs | 89.8 | NG | _[87]_ |
| OA and OAM | 400 nm/100 fs | 970 | NG | _[88]_ |
| DDAB | 400 nm/35 fs | 192 | NG | _[89]_ |
| OLa | 400 nm/100 fs | 60.9 | NG | _[90]_ |
| OA and OAM | 400 nm/50 fs | 102.0 | NG | _[91]_ |
| OctAm and OAc | 800 nm/100 fs | 850 | 569.7 ± 6 | _[92]_ |
|  | 400 nm/150 fs | 14 | 13.9 ± 1.3 |  |
| OA and OAM | 400 nm/100 fs | 5.3 | 450±30 | _[23]_ |
| OA and OAM | 400 nm/50 fs | 95.8 | NG | _[93]_ |
| OA and OAM | 400 nm/120 fs | 550 | 343 ± 6 | _[94]_ |
|  |  | 250 | 321 ± 6 |  |
|  |  | 100 | 309 ± 4 |  |
| lecithin | 400 nm/50 fs | 50.1 | 287.1±7.2 | This work |

Notes

OctAm: n-octylamine

OAc: oleic acid

**Fig. S37.** Gain measurement of the lecithin-PeQD film under ns pulsed laser excitation.

**Fig. S38.** Gain measurement of the lecithin-PeQD film under fs pulsed laser excitation.

**Fig. S39.** (a) ASE emission spectra from lecithin-PeQDs film excited by ns laser pulses for 120 min. (b) ASE emission spectra from lecithin-PeQDs film excited by fs laser pulses for 60 min.

**References**

[1]	M. Li, J. Wang, J. Yao, S. Wang, L. Xu, J. Song, *Adv. Funct. Mater.* **2023**, *34* (3), <https://doi.org/10.1002/adfm.202308341>.

[2]	Z. Gao, X. Shao, Z. Huang, Q. Xie, Y. Ying, H. Lin, J. Wang, X. Tang, W. Chen, W. Pei, Y. Tu, Y. Liu, *Appl. Phys. Lett.* **2024**, *124* (4), <https://doi.org/10.1063/5.0176754>.

[3]	Y. Liu, Q. Xie, Y. Ying, Z. Gao, X. Shao, W. Xia, M. Zhou, W. Pei, X. Tang, Y. Tu, *Chem. Eng. J.* **2024**, *498*, <https://doi.org/10.1016/j.cej.2024.155515>.
